# The Epigenetics of the Endocannabinoid System

**DOI:** 10.3390/ijms21031113

**Published:** 2020-02-07

**Authors:** Rosaria Meccariello, Antonietta Santoro, Stefania D’Angelo, Rossella Morrone, Silvia Fasano, Andrea Viggiano, Riccardo Pierantoni

**Affiliations:** 1 Dipartimento di Scienze Motorie e del Benessere, Università di Napoli Parthenope, via Medina 40, 80133 Napoli, Italy; stefania.dangelo@uniparthenope.it (S.D.); rossella.morrone@studenti.uniparthenope.it (R.M.); 2 Dipartimento di Medicina, Chirurgia e Odontoiatria “Scuola Medica Salernitana”, Università degli Studi di Salerno, Via S Allende Baronissi, 84081 Salerno, Italy; ansantoro@unisa.it (A.S.); aviggiano@unisa.it (A.V.); 3 Dipartimento di Medicina Sperimentale, Università della Campania L. Vanvitelli, via Costantinopoli 16, 80138 Cord Napoli, Italy; silvia.fasano@unicampania.it (S.F.); riccardo.pierantoni@unicampania.it (R.P.)

**Keywords:** endocannabinoids, endocannabinoid system, epigenetics, Δ^9^THC, DNA methylation, histone modifications, non coding RNA, reproduction, spermatozoa

## Abstract

The endocannabinoid system (ES) is a cell-signalling system widely distributed in biological tissues that includes endogenous ligands, receptors, and biosynthetic and hydrolysing machineries. The impairment of the ES has been associated to several pathological conditions like behavioural, neurological, or metabolic disorders and infertility, suggesting that the modulation of this system may be critical for the maintenance of health status and disease treatment. Lifestyle and environmental factors can exert long-term effects on gene expression without any change in the nucleotide sequence of DNA, affecting health maintenance and influencing both disease load and resistance. This potentially reversible “epigenetic” modulation of gene expression occurs through the chemical modification of DNA and histone protein tails or the specific production of regulatory non-coding RNA (ncRNA). Recent findings demonstrate the epigenetic modulation of the ES in biological tissues; in the same way, endocannabinoids, phytocannabinoids, and cannabinoid receptor agonists and antagonists induce widespread or gene-specific epigenetic changes with the possibility of trans-generational epigenetic inheritance in the offspring explained by the transmission of deregulated epigenetic marks in the gametes. Therefore, this review provides an update on the epigenetics of the ES, with particular attention on the emerging role in reproduction and fertility.

## 1. Introduction

The endocannabinoid system (ES) is a complex cell-signalling system identified in the early 1990s following studies on the phytocannabinoid Δ^9^-tetrahydrocannabinol (Δ^9^THC), the main psychoactive constituent of the marijuana plant *Cannabis sativa* [1]. It is widely distributed in biological tissues and is involved in many physiological activities such as pain control, motor functions, thermogenesis, sleep/wake cycle, learning and memory, synaptic plasticity, emotional (mood) regulation, stress response, food intake, inflammatory response, lipid and glucose metabolism, heart function, successful gametogenesis, and reproduction, amongst others [2]. The impairment of ES activity has been linked to several pathological conditions, from behavioural, neurological, and metabolic disorders to infertility and cancer, emphasizing the relevance of the pharmacological modulation of this system for the preservation of health status and the treatment of diseases [2,3]. Indeed, the high expression of the ES in brain areas playing a key role in conditioning processes such as drug-seeking behaviour, smoking, and alcohol addiction, emphasises that the ES is widely sensible to environmental epigenetic cues [4,5,6].

In this respect, epigenetics can be defined as the overall biological processes changing gene expression without any change in the nucleotide DNA sequence [7]. This occurs through the chemical modification of DNA and histone protein tails or through the specific production of regulatory non-coding RNA (ncRNA) [8]. The epigenetic signature is first defined in the embryo during development and cell differentiation, and is remodelled during the life course as a direct consequence of lifestyle and environment with impact on health or disease status [9,10]. Therefore, nutritional status, diet, alcohol addiction, physical activity, stress, and exposure to pollutants, pesticides, or endocrine disruptors, among other aspects, can epigenetically affect gene expression. Epigenetic marks can be delivered among tissues within exosomes, extracellular vesicles, or microvesicles, suggesting the existence of new communication routes in which the products of specific cell types may affect gene expression in different target tissues [11,12,13]. However, recent evidence revealed the transfer of epigenetic marks from gametes to the embryo [14,15] with three possibilities of epigenetic inheritance: (1) cross-generational effects or intergenerational inheritance, when the F1 generation is affected as a consequence of in utero or paternal exposure to environmental cues; (2) multigenerational inheritance when F1 and F2 generations are affected; and (3) trans-generational effects when more than three generations stably present the phenotype caused by epigenetic changes [16].

Therefore, the aim of this review is to provide an update on the epigenetic modulation of the ES and the possible ES-dependent epigenetic effects on gene expression, focusing on the emerging role of the ES in male reproduction and fertility.

## 2. ES in Summary

In 1964 Gaoni and Mechoulam identified Δ^9^THC as the main biologically active constituent of marijuana plant [17] giving the start to a number of studies aimed at identifying its biological targets and the related mechanisms. Hence, in the 1990s the identification of an endogenous ES composed of ligands, receptors, biosynthetic and hydrolysing enzymes, and possible membrane transporters led to the design and synthesis of high-affinity molecules to differentially modulate this endogenous signalling system [1,2,18].

The main endogenous cannabinoids (“endocannabinoids”), anandamide (AEA) and 2-arachydonoylglicerol (2AG), are derivatives of the *n-6* poly unsaturated fatty acid (PUFA) arachidonic acid (ARA), and share some of the effects of Δ^9^THC [1]. They bind the main endocannabinoid receptors, the central type 1 and the peripheral type 2 cannabinoid receptors (CB1 and CB2), which are membrane G-coupled receptors. AEA has also intracellular binding sites for the cationic channel type 1 vanilloid receptor (TRPV1) and for the nuclear peroxisome proliferator-activated receptor γ (PPARγ), whereas 2-AG binds to specific γ-aminobutyric acid (GABA) receptor A subtypes in neuronal cells [1,2,18]

Apart from endocannabinoid biosynthesis, which is mediated by the N-acyl-phosphatidylethanolamine-specific phospholipase D (NAPE-PLD) and the *sn-1*-diacylglycerol lipases (DAGLα and DAGLβ) for AEA and 2-AG, respectively, the key feature of endocannabinoid signalling is the modulation of endocannabinoid tone. In this respect, the fatty acid amide hydrolases (FAAH1 and FAAH2) preferentially hydrolyse AEA (and 2-AG to a lesser extent), while the monoacylglycerol lipase (MAGL) is particularly active in hydrolysing 2-AG [19].

On the basis of unexpected evidence for intracellular reservoirs and transporters of endocannabinoids, the classical “dogma” that endocannabinoids—and in particular AEA—are synthesized and released on demand via hydrolysis of cell membrane phospholipid precursors has been revisited as recently reviewed [20]. AEA can be stored in lipid droplets (adiposomes) in association with FAAH1 and FAAH2, and intracellular AEA transporters have been found in different cell types. These transporters shuttle AEA in several cell districts including the nucleus for binding to PPARs (i.e., the fatty acid binding protein 5 (FABP5)) [21], the endoplasmic reticulum for FAAH-dependent degradation, adiposomes for accumulation or degradation or oxidation, the mitochondrion for oxidation, and lysosomes for degradation ([20] and references therein). Among others, intracellular AEA transporters include the potentially sterol carrier protein 2 [22] and the FAAH-like AEA transporter (FLAT-1), a cytosolic variant of FAAH1 that lacks amidase activity but bounds AEA, facilitating its translocation [23].

Currently, the ES represents a key signalling pathway involved in the modulation of most biological functions [1,2,18]. As a consequence, the pharmacological intervention of the ES components may represent a promising strategy for the management and the treatment of diseases, whereas the interference in ES signalling following phytocannabinoid abuse or the impairment of the system may represent a threat for the maintenance of health status.

## 3. Epigenetic Mechanisms: A Brief Overview

The environmental-dependent modulation of gene expression usually occurs at transcriptional, post-transcriptional, and translational levels. In general, the main epigenetic mechanisms involve the chemical modification of DNA and histone tails with consequences on chromatin architecture and accessibility to transcriptional factors and the production of specific regulatory ncRNA [8].

The main chemical modification of DNA is the covalent transfer of the methyl group (-CH_3_) to cytosine located within cytosine–phosphate–guanine (CpG) islets in the gene promoter region, thus forming 5-methylcytosine (5mC). This modification changes the chromatin structure from an opened (transcriptionally active) to closed (transcriptionally inactive) state [24]. DNA methylation is typically erased during zygote formation to be newly established in the developing embryo in order to address proper embryo development and to drive gene imprinting, the process causing genes to be expressed from a parent of origin-specific manner [25]. Different DNA methyltransferases (DNMTs), the enzymes involved in this epigenetic modification, are classically responsible for de novo and maintaining methylation, but cooperative activity has also been reported and reviewed [26]. Classically, de novo methylation is established by DNMT3A and DNMT3B in participation with DNMT3L, a DNMT devoid of catalytic activity, but capable of assisting de novo methylation, increasing the ability of DNMTs to bind to the methyl group donor, S-adenosyl-L-methionine (SAM). Once established, DNA methylation status is maintained by DNMT1. Conversely, the Ten–eleven translocation methylcytosine dioxygenases (TET1-3) catalyse the oxidation of 5mC to 5-hydroxymethylcytosine (5hmC) [27].

The tight or loose interaction of DNA with basic histone and non-histone proteins deeply affects chromatin structure with consequences for gene expression. The tightly folded part of the chromatin, heterochromatin, is usually transcriptionally inactive, whereas, the loosely folded part of the chromatin, euchromatin, is the site of DNA transcription. At present, nine post-translational modifications occurring at histone protein tails have been identified. The most well-studied are: acetylation (ac), mono- (m1), di- (m2), and tri-methylation (m3) at lysine (K) residues (i.e., the transcription-activating acetylation of histone H3 at lysine 9 or 27 (H3K9ac and H3K27ac, respectively) and the acetylation of histone H4 at lysine 16 (H4K16ac), the transcription activating H3K4me1, H3K4me3, H3K36me3, H3K79me2, and the repressive H3K27me3 and H3K9me3), phosphorylation, and ubiquitination. Crotonylation, citrullination, ADP-ribosylation, proline (P) isomerisation (i.e., H3P30 and H3P38), and *O*-linked β-d-*N*-acetylglucosaminylation (O-GlcNAcylation) are less well known [28,29,30].

NcRNAs virtually influence every aspect of gene expression, thus representing emerging epigenetic marks detectable in both tissues and biological fluids with upcoming relevance in the regulation of biological functions, impact on health and disease status and possible employment for the prognosis, diagnosis and treatment of diseases [31,32]. They include microRNA (miRNA), transfer RNA (tRNA) fragments (tiRNA and tRF), long non-coding RNA (lncRNA), P-element induced wimpy testis (PIWI)-interacting RNAs (piRNAs), short interfering RNA (siRNA), and circular RNA (circRNA) [32,33].

MiRNAs (20–22 nt long) are endogenous small ncRNA classically involved in RNA interference and their exogenous counterparts are siRNAs. In most cases, they bind the 3’ untranslated region of target mRNA inhibiting their translation into protein and inducing their degradation; however in the nucleus miRNAs may target mRNA co-transcriptionally, recruiting chromatin-modifying enzymes and inducing epigenetic regulation via DNA methylation or histone tails modifications [34].

Transfer RNAs (tRNAs) produce several fragments involved in repression of translation. Some of them, called 5’- (3’-) tRNA halves (tiRNA, 30–40 nt long), are stress-induced and are produced in humans by the endonuclease angiogenin that cleaves within the anticodon loops of mature tRNAs. Another group (17–26 nt long), usually referred as tRFs, is produced by the processing at the 5’- or 3’-end of mature or precursor tRNAs [35,36].

LncRNAs are bidirectional, antisense, intronic, intergenic, or overlapping transcripts capable of modulating the transcription of neighbouring protein-coding genes with remarkable tissue specificity. They also remodel chromatin and genome architecture or stabilize RNA through the recruitment of chromatin-modifying enzymes or directly acting as *cis/trans* scaffolding factors [37].

Originally characterized in germ cells, piRNAs (26–31 nt long) target heterochromatic regions through the formation of a PIWI–piRNA complex which usually is associated with the repressive histone/lysine methylation marks, but may also recruit different chromatin-modifying enzymes or facilitate transcription [33].

Lastly, circRNAs, a novel class of ncRNAs, are the result of back-splicing and usually are characterized by a covalently closed continuous loop without 5′ or 3′ polarities structure. Highly stable and widely expressed in mammalian cells, including spermatozoa [38], they usually modulate gene expression by acting as miRNA sponges [39].

Once established at embryo stage to define cell fate through the restriction of developmental potential [8], during the course of life genome activity is dynamically modulated under exogenous influence with gene activation or silencing during the life. Therefore, the continuous interaction between the internal and external environment addresses physiological development and health maintenance influencing both disease load and resistance [40]. Hence, there is hypothesis of an epigenetic “clock” phenomenon, a potential tracker of biological age, in which the aging-dependent genome-wide DNA hypomethylation leads to genome instability and occurrence of disease [41]. As a consequence, various epigenetic writers, readers, and erasers like maintenance and de novo DNMTs, TET proteins, histone acetyltransferases (HATs), deacetylases (HDACs), methyltransferases (HMT), and demethylases (amino oxidase homolog lysine demethylase 1 (KDM1) and JmjC domain-containing histone demethylases), or the ncRNA biosynthetic pathways have been identified in living organisms [32] and their activity is strongly related to the preservation of health status. Epigenetic modifications are linked to changes in development and behaviour, cancer, aging-related diseases, infertility, cardiovascular, neurological and metabolic disorders, or drug addiction, among others [42]. Hence, the development of drugs targeting epigenetic machinery represents the first step for the possible employment of personalized epigenetic therapy in the treatment of diseases [43].

## 4. The Epigenetics of ES

Recent evidence has revealed that ES undergoes epigenetic modulation by alcohol, diet, stress, smoking, exercise, or drugs [44,45,46,47,48,49,50,51,52,53,54,55]. The main targets appear to be the genes encoding for cannabinoid receptors, especially *CNR1* which encodes for CB1*,* and the hydrolysing enzyme *FAAH*, with subsequent alteration of endocannabinoid signalling or tone. The detected epigenetic mechanisms involve changes in DNA methylation (both global and gene-specific), histone tail modifications such as acetylation, deacetylation, or methylation, and the production of specific miRNAs in different brain regions, peripheral tissues, and cell lines. Of note, epigenetic changes in the ES have been detected in several pathological situations such as Alzheimer’s disease, glioblastoma, and colorectal cancer (CRC), and the ES is the target of several ncRNAs [56,57,58,59,60,61,62,63,64], (details in Table 1).

**Figure 1:**
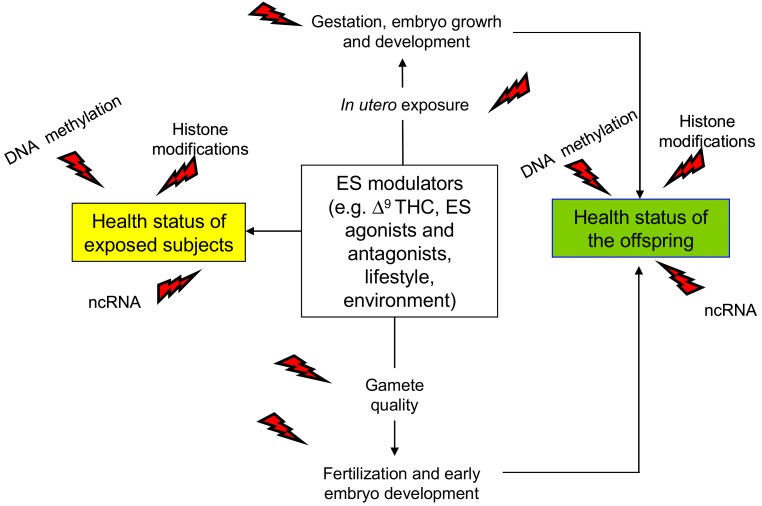
Schematic representation of the main outcomes of endocannabinoid system (ES) epigenetic modulation. Direct effects are in yellow box; trans-generational effects occurring via gametes or following in utero exposure are in green box. ncRNA: non-coding RNA; Δ^9^THC: Δ^9^-tetrahydrocannabinol, red flash of lightning indicates the epigenetic changes.

Nevertheless, phytocannabinoids, endocannabinoids, and endocannabinoid receptor agonist/antagonists all affect epigenetic mechanisms in cell lines, animal models, and humans as well [65,66,67,68,69,70,71,72,73,74,75,76,77,78,79,80,81,82,83,84,85,86,87,88,89,90,91,92,93,94,95,96] (details in Table 2), with a long-term impact on health status and the possibility of transmission to the offspring through gametes, leading to trans-generational epigenetic inheritance (Figure 1). Thus, the ES may represent a potential epigenetic target for the assessment of health/disease status, the treatment of disease, and the development of possible epigenetic therapies.

### 4.1. Effects on Peripheral Tissues, Brain Functions, and Disease State

Peripherally, the activity of ES may be epigenetically modulated by diet. In fact, extra-virgin olive oil (EVOO), a typical lipid source in the Mediterranean diet which is rich in phenolic compounds, epigenetically modulates the expression rate of *CNR1* in vivo and in vitro [51]. Thus, dietary EVOO administration reduces the methylation status of rat *CNR1* promoter and the expression of *miR23A* and *miR-301a*—two modulators of CB1 in the pathogenesis of colorectal cancer, thus inducing the selective expression of CB1 in rat colon. Accordingly, in Caco-2 cells CB1 is less expressed than normal colon mucosa due to the hypermethylation of DNA at *CNR1* promoter; in vitro EVOO, its phenolic extract (OPE), and authentic hydroxytyrosol (HT) upregulate CB1 expression with mechanisms for OPE and HT involving the reduction of *CNR1* methylation at promoter level and leading to inhibition of cell proliferation [51].

In the brain, ES is involved in the homeostatic regulation of food intake, through the interplay with peripheral nutrient-sensors and the orexigenic and anorexigenic peptides produced within the arcuate nucleus of the hypothalamus, the brain region capable of capturing and integrating environmental cues with outcomes on feeding behaviour and reproduction [97]. To date, endocannabinoids act as orexigenic factors, stimulating food intake and fat deposition [97]; consistently, rimonabant (SR141716, Acomplia), the selective CB1 antagonist used in clinical trials for the treatment of obesity, reduces body weight, but due to severe psychiatric side effects, its use in patients has been discontinued [98].

Endocannabinoid are *n-6* PUFA derivatives and an ideal ratio of 5 (*n-6*): 1 (*n-3*) has been suggested in order to preserve brain functions [97]. The evaluation of the plasma *n6:n3* fatty acid ratio is therefore a possible risk factor to metabolic disease and might indicate an over activation of endocannabinoid signalling. Leptin, an anorexigenic peptide produced by white adipose tissue, inhibits hypothalamic ES [99], and *Ob*/*Ob* mice, lacking leptin, over-activate the hypothalamic endocannabinoid signalling [99] and are affected by infertility due to hypogonadotropic hypogonadism [100]. Consistently, leptin resistance has been associated to the over-activation of endocannabinoid signalling, with alterations in food intake and obesity development by a molecular mechanism involving the activation of CB1 [99]. Recently, sex-specific epigenetic changes related to leptin and endocannabinoid signalling have been reported in the hypothalamus of newborn rats following maternal high-fat diet HFD [52]. Prior obesity development, maternal HFD selectively induces the expression of CB1 in the hypothalamus of males, and of CB2 in females, with the former involved in the control of food intake and the latter mainly exerting a neuromodulatory role. Following maternal HFD, the hypothalamic expression of the transcriptional factor STAT3—a signalling intermediate in the leptin-dependent downregulation of the central ES—is down regulated in all newborns, but Almeida and co-workers reported sex-specific mechanisms in the leptin/ES interplay. In fact, while hypoleptinaemia occurred in newborn male rats only, in female rats only a decreased phosphorylation of STAT3 was observed. Thus, two complementary mechanisms impair leptin signalling, leading to the over expression of CB1. Furthermore, in male offspring maternal HFD causes *CNR1* overexpression, with mechanisms involving chromatin remodelling at *CNR1* promoter region by means of increased histone acetylation rate and increased binding of androgen receptor at *CNR1* promoter as a consequence [52].

At present, the *FAAH* gene is the only component of ES epigenetically modulated in the hypothalamus by binge-eating, a recurrent process potentially influencing the development of eating disorders. An epigenetic mechanism consisting in the reduction of H3K4ac, without any change in DNA methylation and H3K27met3 at *FAAH* promoter region have been reported [45].

Interestingly, a highly conserved regulatory sequence in *CNR1* intron 2 is responsible for the differential transcriptional activation of CB1 in brain regions like the hippocampus and hypothalamus, with effects on the sex-specific anxiety-related behavioural profile, ethanol intake, and hypothermic response following CB1 agonism, but without any significant changes in feeding patterns [101].

Lastly, Jiang and co-workers proposed a central adiponectin-dependent mechanism to promote the peripheral bone formation through the epigenetic regulation of the hypothalamic expression of CB1, requiring HDAC5 binding to the transcription start site 2 (TSS2) region of the *CNR1* gene in embryonic mouse hypothalamus cell line N1 [83].

### 4.2. Effects on Male Reproduction and Embryo Development

Virtually all steps of reproduction are affected by one or more elements of the ES. In fact, this signalling system is deeply involved in the central and local control of reproduction in both sexes, with functions related to the modulation of the hypothalamus–pituitary–gonad (HPG) axis, germ cell development, successful gametogenesis, production of high-quality gametes, fertilization, embryo implantation and growth, pregnancy, and delivery [18,102,103,104,105,106,107,108,109]. Centrally, ES regulates the hypothalamic release of gonadotropin releasing hormone (GnRH) which in turn mediates the discharge of pituitary gonadotropins (follicle stimulating hormone (FSH) and luteinizing hormone (LH)), the hormones responsible for sex steroid biosynthesis in the gonads [105,106,107,108]. Nevertheless, the full ES has been characterized in mammalian and non-mammalian vertebrates. Endocannabinoids are produced and hydrolysed within the gonads and reproductive tissues, and are released in reproductive fluids, whereas somatic cells in the gonads, germ cells, and gametes in both sexes have the ability to respond to endocannabinoids [102,105,106,107,108,109,110,111,112,113,114,115,116,117,118,119,120,121,122,123,124]. Lastly, experimental evidence revealed the need for a suitable gradient of endocannabinoids in reproductive tracts to modulate key steps in reproduction such as the acquisition of sperm motility in the epididymis, acrosome reaction, successful embryo implantation, and delivery, among others [102,105,106,125].

**Figure 2:**
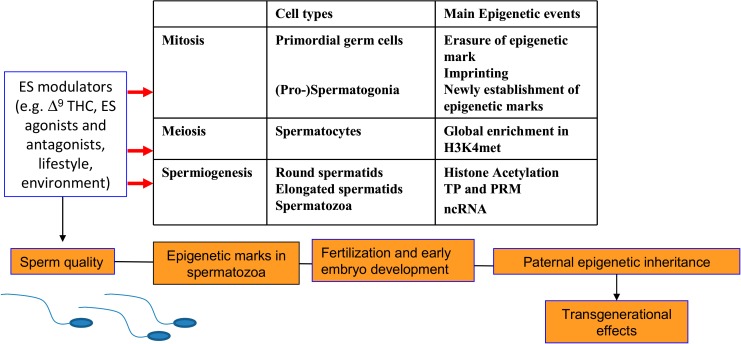
A summary of the main epigenetic changes occurring during the spermatogenesis and the effects of ES modulation. PRM: protamine; TP: transition protein; ncRNA: non-coding RNA.

Reproduction is a process highly sensitive to environmental factors like diet, stress, or endocrine disruptor exposure among others [32,126]. Thus its epigenetic modulation has been reported and reviewed elsewhere [32,126,127,128]. At present, there is a knowledge gap on the possible epigenetic regulation of ES in the modulation of GnRH pulse and reproductive hormones, but, as reported in Section 4.1, the epigenetic modulation of hypothalamic ES has been recently reported in relationship to diet and nutritional status, conditions notably affecting reproductive ability [129]. On the contrary, the epigenetic modulation of ES has been reported in male gonads, with emerging roles in spermatozoa and consequences for fertility and embryo development (Figure 2). Therefore, we will deeply analyse the epigenetic modulation of the ES in the testis.

The first evidence of the epigenetic modulation of ES in the gonad concerns the expression of *FAAH1* in Sertoli cells, the nurse cells within the testis whose survival depends on AEA tone, FSH, and oestradiol activity [130]. The *FAAH1* gene promoter contains an oestrogen-responsive element (ERE) and is notably expressed under oestradiol control in primary mouse Sertoli cells [131]. However, the oestradiol-dependent transcriptional activation of *FAAH1* requires not only the binding of ERβ to proximal ERE sequences (ERE2/3), but also the involvement of the histone demethylase LSD1, and decreased methylation of both DNA at the CpG site and H3K9 in the proximal promoter region [132].

The ES deeply affects the development and the activity of Leydig cells [111], but at present there is no evidence of the possible epigenetic modulation of ES in Leydig cells.

In spite of the lack of data on the possible epigenetic modulation of testicular somatic cells, new insights concern the possible ES-dependent modulation of spermatogenesis through epigenetic mechanisms. Germ cell development is deeply modulated by epigenetic mechanisms which first erase the epigenetic signature (DNA methylation and histone tail remodelling) in primordial germ cells (PGCs) from specifications to migration and proliferation, and subsequently resettle DNA methylation status with de novo development of epigenetic marks and gene imprinting in pro-spermatogonia [9,10]. During post-natal testis development, additional reprogramming of epigenetic marks occurs in two particular time frames: at the entry in meiosis—a process requiring CB2 activity [112,122]—and in post meiotic stages, notably under CB1 control [117,120]. Lastly, during the transit in male and female reproductive tracts, spermatozoa need ES activity for the acquisition of motility and capacitation, respectively [118,125], and this occurs through a deep remodelling process which includes, among others, the exchange/acquisition of such epigenetic marks as ncRNA through epidydymosomes, prostasomes, or oviductosomes [133,134,135,136] that integrates the marks already available in spermatozoa [38,137,138].

In detail, during the spermatogenesis in rodents, CB2 exerts a pivotal role in meiosis entry [112,122] and its hyper- or hypostimulation disrupts the temporal dynamics of the spermatogenesis with possible epigenetic mechanisms [92]. In fact, JWH-133, a CB2 specific agonist, stimulates the expression of the meiotic genes *c-Kit* and *Stra8* by increasing and decreasing the levels of H3K4m3 and H3K9m2, respectively, in genomic regions flanking the transcription start sites. Interestingly, the global increase in H3K4m3 occurs through the JWH-133-dependent transcriptional activation of *Prdm9*, the gene encoding for a zinc finger protein with HMT activity that catalyses H3K4me3 during the meiotic prophase. As a consequence, prolonged exposure to JWH-133 or administration of the specific CB2 antagonist AM630 accelerates or delays spermatogenesis onset in immature mice, pointing out the importance of correct endocannabinoid signalling for proper spermatogenesis and the deleterious effect of exogenous cannabinoids on male fertility [92].

In this respect, the modulation or the interference in the endogenous ES may affect gamete quality or impact the epigenetic mark of gametes, both critical for pregnancy and embryo development, with the (remote, but real) possibility of trans-generational inheritance in the offspring.

Spermiogenesis is the process leading to the formation of spermatozoa and is characterized by round spermatid elongation, acrosome and tail formation, nuclear shaping, and DNA packaging with transcriptional silencing as a consequence. Chromatin remodelling and DNA packaging are therefore the main nuclear events in spermiogenesis, consisting of a double-step process that requires histone replacement, first by transition proteins (TP2 and TP1) and then by protamines (PRM1 and PRM2), a class of small basic proteins [139]. The cooperation between HATs, HDACs, molecular chaperones, ubiquitination, and DNA repair systems drives the shift from a nucleosomal-based to a mainly protamine-based chromatin configuration [139]. In this respect, data from CB1*^-/-^* mice revealed the requirement of ES signalling for proper chromatin remodelling during spermiogenesis [117], and production of high-quality spermatozoa [140]. In fact, the genetic ablation of the *CNR1* negatively affects the chromatin packaging, by affecting the content of *TP2* mRNA and reducing histone displacement, with consequences on chromatin condensation and DNA integrity in the spermatozoa [117], which exhibit nuclear size elongation [140]. Such a mechanism is reversed by oestradiol administration, a treatment promoting histone displacement and chromatin condensation rescue in epididymal sperm collected from knock down animals [120].

In line with the results reported above, recent data revealed that the chronic administration of JWH-133 reduces sperm count in mouse and affects the epigenome of spermatozoa. Interestingly, the sperm from JWH-133 treated mice maintains the ability to fertilize eggs from untreated females, but impairment of embryo growth and defects in placental size have been reported, suggesting a possible interference in paternal inheritance through epigenetic mechanisms [93]. Accordingly defects in DNA methylation/hydroxymethylation at paternally expressed imprinted genes (i.e., *Peg10* and *Plagl1*) have been reported in the sperm of JWH-133 treated animals and are maintained in placental tissue following fertilization. Thus, CB2 signalling may be critical for the integrity of the epigenome in the sperm, with the possibility of paternal epigenetic inheritance in the embryo, a process in which spermatozoa act as vectors for the delivery of epigenetic marks into the developing embryo. Consistently, two isoforms of *CircNAPE-PLD* (*CircNAPE-PLD1* and *CircNAPE-PLD2*) are expressed in human and murine spermatozoa, and *CircNAPE-PLD1* physically interacts with oocyte miRNAs involved in the progression of cell cycle [38]. Therefore, a new role of ES in the zygote to regulate the first stages of embryo development, through epigenetic paternal inheritance aimed at miRNA decoy, has emerged.

The above observations point out the possible risk for epigenome integrity of spermatozoa following marijuana use. Spermatozoa contain a complete ES devoted to the control of sperm physiology; acrosome reaction, acquisition of motility, spermatozoa–oocyte interaction all require the physiological activity of the endogenous ES [105,106]. Classically marijuana smokers exhibit a large set of reproductive failures, from imbalanced hormonal milieu and poor sperm quality to impairment of menstrual cycle, poor oocyte retrieval rate, low pregnancy rate, pre-term delivery, and prematurity with low fetal birth weight [102]. As a consequence, a recent study from the group of Murphy has been focused on the possible epigenetic effects of marijuana smoking and Δ^9^THC on reproductive health status. In humans and rats, Δ^9^THC exposure lowers sperm concentration and alters DNA sperm methylome with substantial shifts in both hypo- and hyper-DNA methylation, with the latter predominating [80]. In particular, 10.3% of differentially methylated CpG sites (409/3979) significantly correlate with sperm count and 183 individual CpG sites representing 177 named genes have methylation levels significantly correlated with measured Δ^9^THC levels [80]. Altered CpG sites associated with genes involved in the Hippo signalling pathway and in pathways in cancer are common in both cannabis users and Δ^9^THC-exposed rats [80]. Interestingly, Δ^9^THC target genes in rat sperm substantially overlap with genes having altered methylation rate in the brain of rat offspring born to parents both exposed to Δ^9^THC during adolescence [79].

Consistently with the above observation, Δ^9^THC exposure-dependent changes in the DNA methylation of rat sperm [80] do not significantly impact the clinical health of the offspring (e.g., litter size, sex ratio, pup birth weight, survival, and growth) but cause long-lasting neurobehavioral effects in the offspring with impairment in attentional performance [78]. In addition, cannabis use in humans causes in the sperm the hypomethylation at 9 CpG sites located in intron 7 of the autism candidate gene *Discs-Large Associated Protein 2* (*DLGAP2)*, a gene involved in synapse organization and neuronal signalling [80,81]. Similarly, Δ^9^THC exposure in adult rats differently methylated *DLGAP2* gene in spermatozoa [81].

Lastly, IBN Lahmar Andaloussi et al. recently reported the behavioural and epigenetic effects of stress in male rats whose fathers were exposed to cannabinoids during adolescence [95] in the presence or absence of the synthetic CB1 agonist WIN55.212-2. Interestingly, stress exposure induced a significant anxiogenic-like effect but did not affect the episodic-like memory in the offspring of WIN55.212-2-exposed fathers only, with significant increases in global DNA methylation and DNMT1 and DNMTa3 transcription in the prefrontal cortex [95]. Thus, these results suggest that chronic exposure to cannabinoids during adolescence may lead to a trans-generational transfer of stress susceptibility to the offspring through the transfer of epigenetic marks in gametes.

Taken together, the use of cannabis for recreational use may represent a serious risk for both the fertility of marijuana smokers and the health of the offspring.

## 5. Conclusions

The ES is an almost ubiquitous cell signalling system regulating several processes inside cells that are not yet completely understood. From its discovery in 1990s, it was clear that this system could be modulated by both extrinsic (cannabis and its derivatives) and intrinsic (the endogenous ligands) signals. Subsequent studies pointed out that the ES is much more complex than was thought since it is able to cross-talk with many other transduction cell signalling pathways, therefore regulating key biological processes such as cell proliferation and differentiation, synaptic plasticity, gametogenesis, and fertility. From the data herein reported, it emerges that epigenetic modifications of the ES by means of DNA methylation, histone acetylation/deacetylation at the *CNR1*, and *FAAH* genes encoding the CB1 receptor and FAAH hydrolysing enzyme may play a relevant role both in physiological processes regulating (male) fertility and reproduction as well as in disease pathogenesis and progression including cancer. Interestingly, it has been documented that external epigenetic cues such as alcohol induce DNA methylation changes in the mouse model of foetal alcohol spectrum disorder, and the lack of a functional *CNR1* gene protects against ethanol-induced impairments of DNMT1, DNMT3A, and DNA methylation [49]. This suggests that the ES itself may act as an epigenetic signal regulating gene expression. On the other hand, the altered DNA methylation of both GPR55 and CB1 encoding genes, resulting in increased expression of GPR55 and reduced levels of the CB1 in CRC patients [58], also supports the hypothesis that the ES receptors could behave either as tumour promoting or tumour suppressor genes depending on the kind of epigenetics changes they undergo. The ES has been recognized as a strong modulator in the central and local control of reproduction in both sexes. It is involved in the regulation of HPG axis, successful gametogenesis, fertilization, and embryo implantation and development. Therefore, it is conceivable that environmental factors, by epigenetically affecting the ES, could induce adverse effects on reproductive system functions per se or alternatively, change gene expression profile with a transgenerational inheritance in the offspring. Within this context, the emerging literature tags the ES signalling as critical for the integrity of the epigenome in the sperm suggesting the possibility of a paternal epigenetic inheritance in the embryo through a process in which spermatozoa act as vectors for delivering epigenetic marks into the developing embryo, and in our opinion this is a very interesting issue that has to be further studied. To date, most studies have been conducted in experimental models other than humans and the few studies in humans involve a limited number of subjects and do not extend to the offspring. However, the overlapping DNA methylation profiles detected in both human and rat sperm suggest that data in experimental models may be the basis for further investigations and confirmation in human subjects. Similarly, the use of phytocannabinoids or synthetic cannabinoids for therapeutic purposes point out the possible trans-generational transmission of epigenetic marks, revealing the need for particular caution and attention in the field. Finally, epigenetic modifiers of the ES could be also a promising tool to treat eating disorders or manage pathological conditions involving alterations of the ES system. However, the significance of ES epigenetics is still an open question needing deeper investigation to better characterize the real consequences of the epigenetic changes of this intriguing cell signalling system.

## Figures and Tables

**Table 1:** The epigenetic modulation of ES by non-canonical ligands and during the disease state.

Epigenetic Cues		Epigenetic Modification/Target	Experimental Model and Subjects (*n*)	Results	References
***Environ-mental factors/lifestyle***	Alcohol and exercise	↑ DNA methylation	Human saliva (Exercise cohort, *n* = 53; case-control cohort, *n* = 81 drinkers and *n* = 81 controls; drinking cohort, *n* = 281)	CpG sites in the *FAAH* gene show opposite DNA methylation patterns in the drinkers vs. exercise cohort	[44]
	Binge-eating episodes	↓ H3K4ac at the *FAAH* gene promoter	Rat brain	Selective down-regulation of FAAH gene expression in the hypothalamus	[45]
	Δ^9^THC smokers, cigarette smokers and non-smokers	↑ methylation rate of the *CNR1* promoter	Human peripheral blood cells (*n* = 77, 36 with Δ^9^THC dependence, 21 cigarette smokers, and 20 non-smokers)	Mean promoter methylation negatively correlated with CB1 expression levels	[46]
	Chronic unpredictable stress	↓ H3K9ac and ↑ HDAC2 activity	Mice	Reduced expression of NPY and CB1 in the cingulated cortex	[47]
	Ethanol	↑ H4K8ac at *CNR1* exon 1	Postnatal day 7 mice	Increased CB1 expression in the hippocampus and neocortex, causing neurobehavioral abnormalities in adult mice	[48]
	Ethanol	↓ DNMT1 and DNMT3A levels impairing DNA methylation	Mouse model of foetal alcohol spectrum disorder. PD7 wild type and CB1^-/-^ mice	The lack of CB1 rescues the loss of DNMT1, DNMT3A, and DNA methylation	[49]
	Ethanol	↑ histone acetyltransferase activity and ↑ histone H3 acetylation	Murine BV2 microglial cells	Down regulation of *Nape-pld*	[50]
	Extra-virgin olive oil (EVOO)	↓ DNA methylation of *CNR1* promoter ↓ *miR23A* ↓*miR-301a*	Short- and long-term dietary EVOO rats and human colon cancer (CaCo-2) cells	Increased expression of the CB1 and reduced proliferation of colorectal cancer cells	[51]
	Maternal high-fat diet (HFD)	↑ histone acetylation rate	Rat hypothalamus	Chromatin remodelling and increased binding of androgen receptor at *CNR1* promoter leading to over expression of CB1	[52]
***Drugs***	Dex	*miR-665*	Rat heart Langendorff preparation	Heart protective effect against ischemia/reperfusion injury via regulation of AK1 and *CNR2*	[53]
	Epigenetic modifiers (trichostatin A and 5-aza-2′-deoxycytidine)	Differential de novo expression of CB1, CB2 and μ-opioid receptors	Human SH SY5Y neuroblastoma cells and human Jurkat T lymphocytes	Selective de novo induction of CB1, CB2, and μ-opioid receptors depending on cell type	[54]
	17β estradiol	↓ H3K27 ↑ H3 and H4 acetylation	SW620 and DLD1 human colon cancer cells	Increased expression of CB1 by enhancing the binding of oestrogen receptor (ER) α and ERβ to *CNR1* depending on cell type	[55]
***Disease***	Alzheimer’s disease	↓ DNA methylation at *FAAH* gene	Peripheral blood mononuclear cells (PBMCs) from subjects with late-onset Alzheimer disease (LOAD) (*n* = 33) and healthy controls (*n* = 33)	FAAH protein, and activity increased in PBMCs of LOAD subjects	[56]
	Glioblastoma	Differential DNA methylation	Tumour samples (*n* = 55) and non-neoplastic brain tissues (*n* = 5) for methylation analyses; tumor samples (*n* = 40) and control (*n* = 3) for gene expression	Under expression of FAAH with hypermethylated promoter	[57]
	CRC	↑ DNA methylation of *CNR1* at CpGs located from –755 to +268 ↑ *GPR55* demethylation	Human CRC tissues (*n* = 566)	GPR55 is highly expressed in CRC patients while CB1 levels are reduced	[58]
***ncRNA***	*miR-1273g-3p*	↓ CB1	Human colorectal cancer LoVo cell lines	Promotion of proliferation, migration, and invasion	[59]
	*miR-29a*	↓CB1 ↑PPAR-γ	Gain-of-function transgenic mice	Block of the expressions of proinflammatory and profibrogenic mediators; attenuation of renal hypertrophy	[60]
	*miR-494*	CB1	Myocardial biopsy specimens (*n* = 12 chronic heart failure (CHF) and *n* = 4 healthy controls)	In CHF *miR-494* is slightly increased leading to a compensatory response of the diseased myocardium.	[61]
	*miR-665*	CB2	Myocardial biopsy specimens (*n* = 12 CHF and *n* = 4 healthy controls)	In CHF *miR-665* expression is significantly decreased leading to a compensatory response of the diseased myocardium.	[61]
	*hsa-miR-29b-3p*	CB1	Paediatric low-grade gliomas (P-LGG) (*n* = 33) and control brains (*n* = 6)	Spontaneous involution of P-LGG may be induced by endocannabinoids	[62]
	CB1 *hsa-let-7d*	↑ *hsa-let-7d* and other miRNA ↓CB1	Various in vitro and in vivo systems	CB1 receptor up-regulates *let-7d*, which, in turn, impairs CB1 receptor signalling and cannabinoid-opioid cross-signalling.	[63]
	*AntagoMir-411*	↓ miR-411 ↑FAAH ↑Pparδ ↑glutamate receptor AMPA-2	Prefrontal cortex of female C57BL/6J mice	Reversion of alcohol-related neuro-adaptations and reduction of chronic alcohol consumption	[64]

↑ increase; ↓ decrease

**Table 2:** Epigenetic changes induced by phytocannabinoids, endocannabinoids, and ES agonists/antagonists.

Substances	Epigenetic Modification	Experimental Model and Subjects (*n*)	Results	References
***Phytocannabinoids***				
*Cannabis*	Changes in DNA methylation	Blood from schizophrenia patients (*n* = 98) and healthy controls (*n* = 108)	Modulation of the immune response and protection against the neurobiological substrate of reality distortion symptoms in schizophrenia patients	[65]
Cannabidiol Cannabigerol	↑ DNA methylation of *keratin 10* gene	Human keratinocytes (HaCaT cells)	↓*keratin 10* mRNA through a CB1-dependent mechanism, whereas cannabigerol did not affect either CB1 or CB2	[66]
Cannabidiol	↑Global DNA methylation levels ↑*DNMT1* expression No effect on DNMT 3a, 3b, or 3L	HaCaT cells	Modulation of gene repression	[66]
Δ^9^THC	Changes in DNA methylation profile	Non-human primates, brain (lateral cerebellum) during simian immunodeficiency virus infection	Altered gene expression	[67]
Δ^9^THC	Dose-dependent increase of HDAC3 expression	Human BeWo trophoblast cell line	Inhibition of proliferation	[68]
Δ^9^THC	↓H3K9me3 ↓H3K4me3 in the nucleus accumbens shell	**Adolescent rats**	Proenkephalin (*Penk*) upregulation in the adult and opiate vulnerability	[69]
Δ^9^THC	↑H3K9m2 ↓H3K4m3	Rats, prenatal exposure	Decreased dopamine receptor D2 (*Drd2*) RNA expression in the ventral striatum (nucleus accumbens) in adult animals	[70]
Δ^9^THC	Histone modifications (H3K9me2, H3K9me3, H3K27me3, H3K9ac and H3K14ac)	Adolescent and adult brain areas (hippocampus, amygdala and nucleus accumbens) of female rats	Region- and age-specific histone modifications leading to transcriptional repression in the adolescence and transcriptional activation in the adults	[71]
Δ^9^THC	Histone modifications, mainly H3K9me3	Adolescent female rats, prefrontal cortex	Increased expression of the histone-lysine N-methyltransferase SUV39H1 Cognitive deficit	[72]
Δ^9^THC	Histone modifications (H3K4me3, H3K9me3, H3K27me3, H3K36me3 and H3K9ac)	Differentiating lymph node cells of mice immunized with a superantigen, staphylococcal enterotoxin B	Alterations in antigen-specific T cell response due to altered gene expression	[73]
Δ^9^THC	↓ *miR-17/92* cluster ↓ *miR-374b/421* cluster ↑ *miR-146* ↑ LncRNAs expressed from the opposite strand of *CD27* and *Appbp*2	Mouse super antigen-activated lymph node cells and CD4^+^ T cells	Altered transcripts mainly related to immune response and cell proliferation	[74]
Δ^9^THC	Not Assayed	Long-Evans rats with parental Δ^9^THC exposure	Deregulated mRNA levels (i.e., *CNR1*, glutamate and dopamine-related genes) in the striatum of adolescent and adult F1 offspring; behavioural and neurobiological abnormalities in the F1 offspring	[75]
Δ^9^THC	Up-down regulation of several miRNAs	Non-human primates, CD4^+^ T cells, during simian immunodeficiency virus infection	Immunomodulatory role for cannabinoids	[67]
Δ^9^THC	Modulation of miRNAs, including ↑*miRNA-690* and its target Transcription factor CCAAT/enhancer-binding protein α	Mouse myeloid-derived suppressor cells	Altered myeloid expansion and differentiation	[76]
Δ^9^THC	Up/down regulation of several miRNAs like ↑*miR-10a*, ↑*miR-24,* ↑*miR-99b*, ↑*miR-145*, ↑*miR-149*, ↑*miR-187*	Intestine of simian immunodeficiency virus infected macaques	Altered miRNA profile and changes in anti-inflammatory response	[77]
Δ^9^THC	DNA methylation in sperm	Paternal exposure of rats	Long-lasting neurobehavioral effects in the offspring	[78]
Δ^9^THC	1027 differentially methylated regions in F1 adults	Paternal exposure of rats	Cross-generational epigenomic alterations in the rat nucleus accumbens, including differentially methylated regions localized to genes with important roles in neural function, complex psychiatric diseases, and addiction-related traits	[79]
Δ^9^THC	Global DNA methylation	Human (*n*= 24 including 12 cannabis smokers and 12 cannabis non-smokers) and rat sperm	Changes in DNA sperm methylome, with altered CpG sites associated with genes involved in Hippo signalling and cancer pathways	[80]
Δ^9^THC/*Cannabis*	DNA methylation	Humans (*n*= 24 including 12 cannabis smokers and 12 cannabis non-smokers) and paternal exposure of rats	Changes in the methylation of the autism candidate gene *DLGAP2* in human and rat sperm and in the nucleus accumbens of the offspring of the Δ^9^THC exposed rats	[81]
***Endocannabinoids and ES synthetic agonists and antagonists***				
ACEA JWH-133 AM-281 AM-630	↓*miR-23a* ↓*miR-24*, ↓*miR-181a* ↓*miR-320a*	Human granulosa cell line KGN	Modulating role of the intrinsic ovarian ES in the regulation of oestradiol synthesis and alteration in miRNA profile following CB1 manipulation only	[82]
ACEA SR141716A	Enhanced expression levels of HDACs- especially HDAC5- which binds *CNR1*promoter	Embryonic mouse hypothalamus N1 cell line and mouse hypothalamus	Attenuated or enhanced central adiponectin (APN) induction of bone formation	[83]
ACPA SR141716A	Modulation of HDAC activity	Mouse	Combined involvement of histone acetylation and ES system in anxiety- and depression-related behaviours	[84]
AEA	Increased DNA methylation and DNMT activity	HaCaT cells	Inhibition of differentiation	[85]
AEA	609 miRNA differentially regulated	Methylated bovine serum albumin-induced delayed type hypersensitivity response in C57BL/6 mice, mediated by Th17 cells	Altered interleukin production and inflammatory response	[86]
AM-251	Restoration of H3K9ac at control levels	Hippocampus of schizophrenia like animals	ES-dependent epigenetic mechanisms involved in both embryonic brain development and neuro-differentiation as well as in the pathophysiology of a schizophrenia like phenotype	[87]
AM-251	↑*miR-30e-5p* ↓DLL4 in adipose tissue macrophage	F4/80+ cells from stromal vascular fractions of epididymal fat collected from DIO mice fed HFD	Suppression of DLL4-Notch signalling-induced polarization of inflammatory Th1 cells and adipocyte energy storage with anti-inflammatory state and attenuation of DIO phenotype	[88]
FAAH-II	Up-down regulation of several miRNAs, including imprinted *Dlk1-Dio3* miRNA cluster	Mouse mesenteric lymph nodes and Peyer’s patches	Suppression of colitis through regulation of pro-inflammatory miRNA expression	[89]
HU-210	Differential miRNA expression	**Postnatal day** 35 rats	Significant differences in the expression of miRNA in the left hemisphere of the entorhinal cortex, in a manner that is relevant to schizophrenia	[90]
HU-210 JWH-133	↑H3K9me3	Glioma cell lines U87MG and U373MG expressing CBs	Induction of differentiation, inhibition of gliomagenesis	[91]
JWH-133	↑H3K4m3 ↓H3K9m2	Mouse spermatogonia in vitro	Increased expression of the meiotic genes *c-Kit* and *Stra8* with accelerated meiosis entry	[92]
JWH-133	Altered DNA methylation and hydroxymethylation at specific imprinted genes in sperm and placenta	Paternal exposure in mouse	Reduced sperm count in exposed animals and defects in placental and embryonic development	[93]
SR141716A	↓ *miR-466* family ↓ *miR-762* and other	DIO mice fed HFD	Attenuation of DIO-associated inflammation	[94]
WIN55,212-2	Increased expression of DNMTs and DNA methylation in prefrontal cortex	Paternal exposure in rats during adolescence	Increased vulnerability to stress in the offspring	[95]
WIN55,212-2	DNA hypermethylation at the intragenic region of the intracellular signalling modulator *Rgs7*	Adolescent mice	Reduced expression of *Rgs7* in the hippocampus and memory impairment in adult mice	[96]

ACEA: synthetic agonist at CB1; ACPA: synthetic agonist at CB1; AM-251: inverse agonist at CB1; AM-281: CB1 inverse agonist/antagonists; AM-630: inverse agonist/antagonist at CB2; FAAH-II: FAAH inhibitor; HU-210: synthetic cannabinoid agonist; JWH-133: synthetic cannabinoid agonist at CB2; SR141716 (Rimonabant): CB1 inverse agonist; WIN55,212-2: synthetic cannabinoid agonist. ↑ increase; ↓ decrease.

## Abbreviations

ACEA	arachidonyl-2′-chloroethylamine
ACPA	arachidonylcyclopropylamide
AEA	anandamide
2AG	2-arachydonoylglicerol
APN	adiponectin
ARA	arachidonic acid
CB1	type 1 cannabinoid receptor
CB2	type 2 cannabinoid receptor
CHF	chronic heart failure
circRNA	circular RNA
CNR1	gene encoding for CB1
CpG	cytosine–phosphate-guanine
CRC	Colon Rectal Cancer
DAGL	*sn-1*-diacylglycerol lipase
Dex	dexmedetomidine
DIO	diet-induced obesity
DLGAP2	discs large associated protein 2
DNMT	DNA methyltransferases
*Drd2*	gene encoding for dopamine receptor D2
Δ^9^THC	Δ^9^-tetrahydrocannabinol
ER	oestrogen receptor
ERE	oestrogen responsive element
ES	endocannabinoid system
EVOO	extra-virgin olive oil
FAAH1	fatty acid amide hydrolase 1
FAAH2	fatty acid amide hydrolase 2
FABP5	fatty acid binding protein 5
FLAT-1	FAAH-like AEA transporter
FSH	follicle stimulating hormone
GABA	γ-aminobutyric acid
GlcNAcylation	*O*-linked β-d-*N*-acetylglucosaminylation
GnRH	gonadotropin releasing hormone
HAT	histone acetyltransferases
HDAC	histone deacetylases
HFD	high-fat diet
5hmC	5-hydroxymethylcytosine
HMT	histone methyltransferases
HPG	hypothalamus-pituitary-gonad
HT	hydroxytyrosol
KDM1	amino oxidase homolog lysine demethylase 1
LH	luteinizing hormone
lncRNA	long non-coding RNA
LOAD	late-onset Alzheimer’s disease
MAGL	monoacylglycerol lipase
5mC	5-methylcytosine
miRNA	microRNA
NAPE-PLD	N-acyl-phosphatidylethanolamine-specific phospholipase D
ncRNA	non coding RNA
OPE	olive oil phenolic extract
PBMC	peripheral blood mononuclear cells
*Penk*	gene encoding for Proenkephalin
PGC	primordial germ cell
P-LGG	paediatric low-grade gliomas
piRNA	PIWI-interacting RNA
piwi	P-element induced wimpy testis
PPARγ	peroxisome proliferator-activated receptor γ
PRM	protamine
PUFA	poly unsaturated fatty acids
SAM	S-adenosyl-L-methionine
siRNA	short interfering RNA
TET	Ten-eleven translocation methylcytosine dioxygenases
tiRNA and tRF	tRNA fragments
TP	transition protein
TRPV1	cationic channel type 1 vanilloid receptor
TSS	transcription start site
